# Regulation of brain iron uptake by apo- and holo-transferrin is dependent on sex and delivery protein

**DOI:** 10.1186/s12987-022-00345-9

**Published:** 2022-06-10

**Authors:** Stephanie L. Baringer, Elizabeth B. Neely, Kondaiah Palsa, Ian A. Simpson, James R. Connor

**Affiliations:** 1 Department of Neurosurgery, Penn State College of Medicine, Hershey, PA USA; 2 Department of Neural and Behavioral Sciences, Penn State College of Medicine, Hershey, PA USA; 3 Penn State College of Medicine, 500 University Drive, 17033 Hershey, PA United States

**Keywords:** Blood–brain barrier, Iron, H-ferritin, Transferrin, Sex difference

## Abstract

**Background:**

The brain requires iron for a number of processes, including energy production. Inadequate or excessive amounts of iron can be detrimental and lead to a number of neurological disorders. As such, regulation of brain iron uptake is required for proper functioning. Understanding both the movement of iron into the brain and how this process is regulated is crucial to both address dysfunctions with brain iron uptake in disease and successfully use the transferrin receptor uptake system for drug delivery.

**Methods:**

Using in vivo steady state infusions of apo- and holo-transferrin into the lateral ventricle, we demonstrate the regulatory effects of brain apo- and holo-transferrin ratios on the delivery of radioactive ^55^Fe bound to transferrin or H-ferritin in male and female mice. In discovering sex differences in the response to apo- and holo-transferrin infusions, ovariectomies were performed on female mice to interrogate the influence of circulating estrogen on regulation of iron uptake.

**Results:**

Our model reveals that apo- and holo-transferrin significantly regulate iron uptake into the microvasculature and subsequent release into the brain parenchyma and their ability to regulate iron uptake is significantly influenced by both sex and type of iron delivery protein. Furthermore, we show that cells of the microvasculature act as reservoirs of iron and release the iron in response to cues from the interstitial fluid of the brain.

**Conclusions:**

These findings extend our previous work to demonstrate that the regulation of brain iron uptake is influenced by both the mode in which iron is delivered and sex. These findings further emphasize the role of the microvasculature in regulating brain iron uptake and the importance of cues regarding iron status in the extracellular fluid.

## Background

Iron plays an essential role in many important biological functions, including cognition and overall brain health. As an electron donor and acceptor, as well as a carrier of oxygen, iron is vital to cellular metabolism [1]. Furthermore, iron is utilized in both the formation of myelin and the synthesis of many neurotransmitters, such as dopamine and norepinephrine [1]. Fluctuation of optimal iron levels can cause many neurological issues. Low levels of brain iron in adults are connected to Restless Legs Syndrome [2, 3] and sleep disorders [4]. On the other hand, excessive brain iron is linked to many neurodegenerative disorders [5], such as Parkinson’s disease [6], amyotrophic lateral sclerosis [7], and Alzheimer’s disease [8, 9]. The importance of iron homeostasis for neurological health and proper functioning requires tight regulation at the blood–brain barrier (BBB).

Historically it was posited that endothelial cells (ECs) of the BBB, which make up approximately 2% of the brain [10], passively transport iron from blood to brain. The premise was that holo-transferrin (Tf) (iron rich) bound to its receptor, on the luminal membrane and was transcytosed to the abluminal space. However, this model did not consider the iron needs of the ECs nor did it acknowledge the clear need for regulation of iron access to the brain. Our laboratory and others have since demonstrated regulation of iron uptake by ECs [11–16]. Specifically, our group has shown that apo-Tf (iron poor) in the basal space increases both iron transport and release from ECs in vitro [14]. Furthermore, the Tf transcytosis theory does not account for the delivery of iron to the brain by H-ferritin (Fth1), which has gained increasing interest as an iron delivery protein [17–19]. In addition to our group’s previous work exploring the in vitro effects of apo- and holo-Tf, Chiou et al. have suggested that iron uptake into the brain is regulated by ECs, which control uptake into the cells, storage of iron therein, and subsequent release into the brain [14]. In the present study we examined both the uptake of iron within the microvasculature and its subsequent release into the brain parenchyma. Moreover, we hypothesized that sex differences, which have been shown to be prominent in brain iron acquisition [18, 20, 21], would be subject to regulation of iron uptake by apo- and holo-Tf as well as the type of iron delivery protein.

## Methods

### Experimental design

**Fig. 1:**
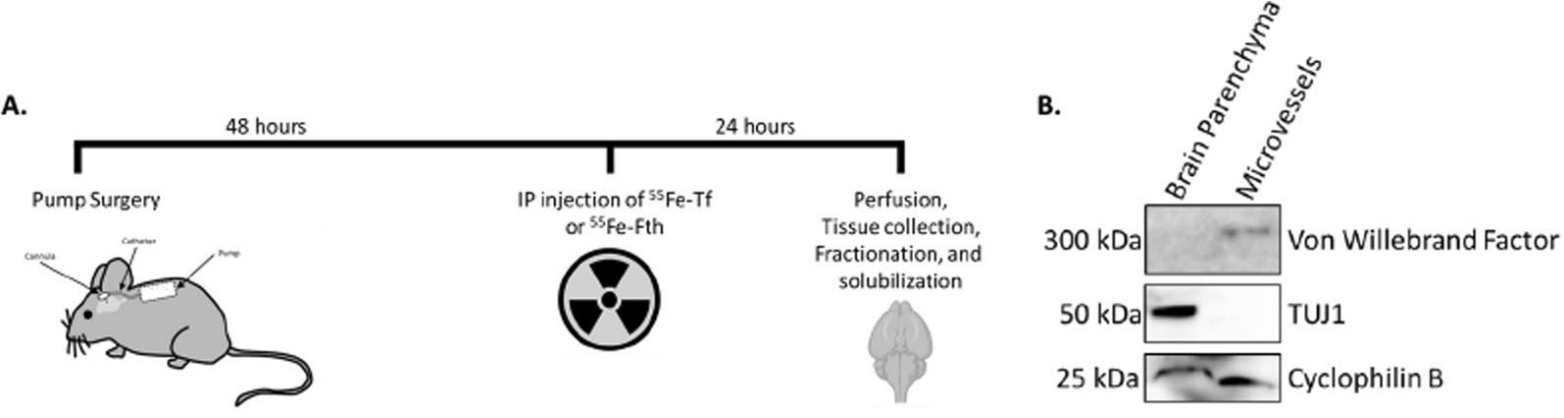
Experimental setup. Mini osmotic pumps were inserted subcutaneously in three-month-old male and female mice (**A**). Pumps contained nothing (sham), aCSF, 1 mg/mL apo-Tf, or 1 mg/mL holo-Tf. Forty-eight hours after the surgery, mice were injected IP with radioactive ^55^Fe-Tf or ^55^Fe-Fth1. Twenty-four hours later the mice were euthanized and perused. Brains were collected and homogenized. Microvessels (MV) were isolated from the brain parenchyma using centrifugation. Both fractions of MVs and brain parenchyma were further solubilized. Radioactivity in each fraction was determined using liquid scintillation counting. Western blotting was performed on brain parenchyma and MV fractions (**B**). The blots show von Willebrand factor, an endothelial cell specific marker, present in the MV fraction and not in the brain parenchyma fraction. TUJ1, a neuronal marker, is shown in the brain parenchyma fraction and not the MV fraction. Cyclophilin B was used as a loading control for samples

Mini osmotic pumps were inserted subcutaneously connected to a cannula inserted in the lateral ventricle (Fig. 1). After 48 h of infusion, animals were injected intraperitoneally with either ^55^Fe-Tf or ^55^Fe-Fth1. Twenty-four hours later, brains were harvested and separated into microvessel and brain parenchyma fractions. The tissue was then solubilized and counted using liquid scintillation counting.

### Pump surgery

Forty-eight hours prior to the surgery, osmotic pumps (infusion rate 0.25 µL/h, Alzet, model 2004) were filled according to manufacturer instructions with nothing (sham), artificial cerebrospinal fluid (aCSF, 125 mM NaCl, 2.5 mM KCl, 1 mM MgCl-6H_2_O, 1.25 mM NaH_2_PO_4_, 2 mM CaCl-2H_2_O, 25 mM NaHCO_3_, 25 mM glucose, pH 7.3), 1 mg/mL apo-Tf in aCSF, or 1 mg/mL holo-Tf in aCSF. Three-month-old wildtype (B6; 129 × 1-Hfetm1Jrco/J background) mice were subjected to pump insertion under isoflurane anesthesia (1–2%). A power analysis revealed n = 5 was required for 80% at alpha 0.05. Briefly, the pump with attached tubing was placed subcutaneously and the cannula was placed 1 mm lateral to Bregma and 0.5 mm posterior to deliver the pump contents directly to the lateral ventricle. This placement was considered sufficient to influence iron uptake by the microvasculature given that the well-established dynamic equilibrium of CSF and interstitial fluid, allowing the pump contents to distribute throughout the brain parenchyma [22]; similar to endogenous Tf produced by the choroid plexus [23]. The inclusion of aCSF as an experimental condition allows us to exclude the vehicle to be the cause of changes and informs us of general infusion effects and to determine any dilution effect of the endogenous Tf on the iron uptake. The incision was then sutured with nylon sutures. The mice were then placed in a heated recovery chamber until they regained consciousness, and accordingly, they were returned to their cages. Mice were maintained under normal housing conditions. They were given ad libitum access to rodent chow pellets and water. Both males and females were included in experiments. This study complies with the ARRIVE 2.0 guidelines. All procedures were conducted according to the NIH Guide for the Care and Use of Laboratory Animals and were approved by the Pennsylvania State University College of Medicine Institutional Animal Care and Use Committee.

### Iron protein preparation

Wild-type human Fth1 containing a poly-His tag was subcloned into pET30a(+), to be produced in BL21 *Escherichia coli* [17]. Isopropyl-β-d-thio-galactoside (IPTG) was used to induce expression. Following this, bacteria were lysed, and Fth1 protein was purified on a nickel column using standard techniques (GE Healthcare Bio-Sciences). Transferrin was purchased commercially (Sigma).

### Radiolabeling

^55^Fe (Perkin Elmer) was complexed with 1 mM nitrilotriacetic acid (NTA), 6 mM ferric chloride (FeCl_3_), and 0.5 M sodium bicarbonate (NaHCO_3_) at a ratio of 100 µL NTA:6.7 µL FeCl_3_:23.3 µL NaHCO_3_:50 µCi ^55^FeCl_3_ to form the ^55^Fe-NTA complex [14]. After complexing, ^55^Fe-NTA was incubated with apo-Tf (Sigma) or Fth1 for 30 min to allow for iron loading. Unbound iron was separated from the total complex using PD midiTrap-G25 columns following manufacturer’s instructions (GE Healthcare Bio-Sciences).

### Uptake studies

Mice received a single intraperitoneal injection of 3.4 mg/kg body weight ^55^Fe-Tf or ^55^Fe-Fth1. 24 h after injection, blood was drawn and mice were transcardially perfused with 0.1 M phosphate-buffered saline (PBS, pH 7.4). Brains were collected, weighed immediately, and homogenized on ice using disposable tissue grinders (VWR) and MVB Buffer (0.147 M NaCl, 0.4 mM KCl, 0.3 mM CaCl_2_, 0.12 mM MgCl_2_, 15 mM HEPES, 0.5% BSA, 5 mM glucose). Homogenates were transferred to microcentrifuge tubes and spun at 1000×*g* for 10 min at 4 °C. The supernatant was collected, and the pellet was resuspended in buffer and spun again. The resulting supernatant was combined with the previous collection and termed brain parenchyma. The pellet was resuspended again and termed microvessels (MVs). Validation of these fractions can be found in Fig. 1. This separation allowed us to determine the amount of ^55^Fe that was released from the MVs and entered the brain or was sequestered in the MVs. Tissue was solubilized using 1 mL Solvable (Perkin Elmer) according to manufacturer’s instructions. After solubilization, 10 mL Hionic-Fluor scintillation cocktail (Perkin Elmer) was added. Samples were counted using the Hidex 300 SL (LabLogic) for three minutes each. Blank tube values were subtracted from final counts to correct for background counts.

### Protein detection

Brain homogenates were spun at 1000×*g* for 10 min at 4 °C [20]. The supernatant (cortical fraction) was spun at 14,000×*g* for 10 min. The resulting cell pellet was resuspended and digested in RIPA buffer (Sigma) containing protease inhibitor cocktail (PIC, Sigma) for 1 h on ice. The MV pellet was resuspended and digested in a mixture of RIPA buffer (Sigma) and protease inhibitor cocktail (PIC, Sigma) for 1 h on ice. All homogenates were sonicated on ice for 90 s and spun at 14,000×*g* for 10 min at 4 °C for final collect of the protein lysate. Total protein was quantified by bicinchoninic assay (BCA, Pierce) and 25 µg was loaded onto a 4–20% Criterion TGX Precast Protein Gel (Bio-Rad). Protein was transferred onto a nitrocellulose membrane and probed for the neuronal marker TUJ1 (Abcam, 1:1000, ab18207) or the brain MV marker von Willebrand factor (Abcam, ab174290, 1:1000) and cyclophilin B as a loading control (Abcam, ab16045, 1:1000). Corresponding secondary antibody conjugated to HRP was used (1:5000, GE Amersham) and bands were visualized using ECL reagents (Perkin-Elmer) on an Amersham Imager 600 (GE Amersham).

### Ovariectomy

Two-month-old female mice were subjected to aseptic bilateral surgical ovariectomy (OVX) via a dorsal incision under isoflurane anesthesia (1–2%). After surgery, the skin was sutured with nylon sutures. These mice were then placed in a heated recovery chamber until they regained consciousness, and accordingly, they were returned to their cages. After 2 weeks, blood was collected from OVX mice and four equally aged intact mice to act as a control.

### Serum molecule detection

Blood was collected via submandibular cheek blood collection in heparin-coated tubes. Serum was separated from whole blood fractions by centrifugation at 2000×*g* for 15 min. Serum levels of estradiol were measured by enzyme-linked immunosorbent assay (Cayman Chemical, 501890) according to the manufacturer’s protocol. Total iron binding capacity (TIBC), transferrin percent saturation, and serum iron were measured using an assay kit (Abcam, ab239715).

### Statistical analysis

Statistical analyses were performed using Prism 9.2 software (Graphpad Software Inc.). Data from at least five independent biological replicates were averaged and are expressed as the mean ± standard deviation (SD). One-way ANOVA with Tukey post-hoc analysis or unpaired t-tests were used to evaluate for statistical significance where appropriate. A p-value < 0.05 was considered significant.

## Results

### ^55^Fe-Tf brain uptake is responsive to apo- and holo-Tf in a sex-dependent manner

**Fig. 2:**
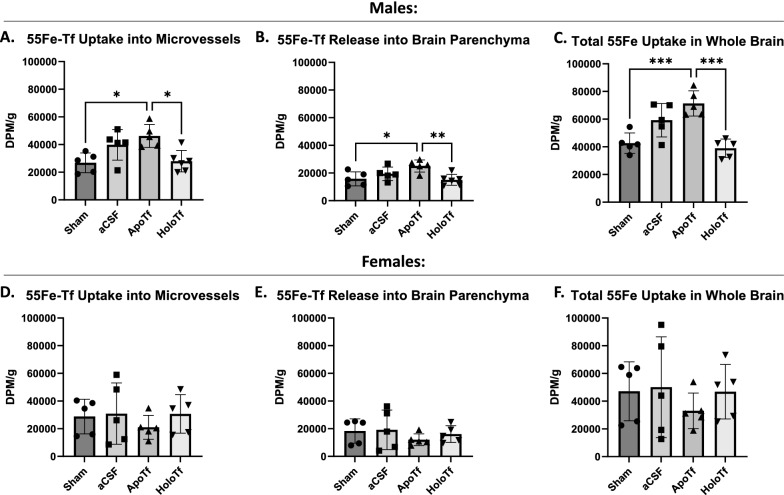
^55^Fe-Tf brain uptake in males and females. Samples are reported as DPM per gram of brain tissue. In males, increasing levels of apo-Tf in the brain significantly increases ^55^Fe-Tf uptake into MVs (**A**) and release into the brain (**B**). Additionally, increasing levels of holo-Tf results in significantly reduced ^55^Fe-Tf uptake compared to infusions of apo-Tf. This difference is further demonstrated when MV and parenchyma fractions were pooled for total uptake in the whole brain (**C**). However, in females, the ratio of apo- to holo-Tf in the brain has little regulatory effect on ^55^Fe-Tf uptake into MVs (**D**), release into the parenchyma (**E**), and total uptake in the whole brain (**F**). n = 5 for all conditions, means of biological replicates ± SD were evaluated for statistical significance using one-way ANOVA with Tukey’s posttest for significance. * p < 0.05, ** p < 0.01, *** p < 0.001

The aim of the first study was to examine the regulatory effects of apo- and holo-Tf on ^55^Fe-Tf uptake. In males, both MV levels of ^55^Fe-Tf (Fig. 2a) and parenchymal levels (Fig. 2b) were significantly increased with apo-Tf infusions (*p < 0.05) by nearly 41%. In contrast, infusion of holo-Tf resulted in levels of ^55^Fe-Tf in both MVs and parenchyma significantly lower than observed with apo-Tf infusion (*p < 0.05 and **p < 0.01). When the MV and brain parenchyma fractions were pooled together to examine whole brain uptake, the effect of apo-Tf on ^55^Fe-Tf uptake is even more apparent (Fig. 2c, ***p < 0.001). The infusion of aCSF increased MV and parenchymal levels of ^55^Fe-Tf, though not statistically significant or to the same level as apo-Tf. However, in females, neither MV, parenchymal, or whole brain levels of ^55^Fe-Tf (Fig. 2d–f), were responsive to apo- or holo-Tf infusions. Notably, about 50% more ^55^Fe-Tf was sequestered in the MVs than was released into the parenchyma, supporting the regulator role of the MVs regardless of sex.

### ^55^Fe-Fth1 brain uptake is not responsive to apo- or holo-Tf

**Fig. 3:**
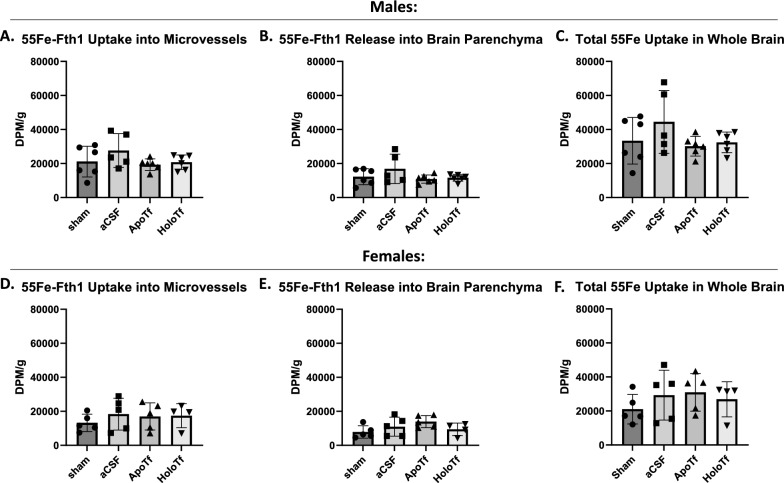
^55^Fe-Fth1 brain uptake in males and females. Samples are reported as DPM per gram of brain tissue. In males, the ratio of apo- to holo-Tf in the brain has little regulatory effect on uptake into MVs (**A**), release into the parenchyma (**B**), or uptake into the whole brain (**C**). In females, the ratio of apo- to holo-Tf in the brain has little regulatory effect on uptake into MVs (**D**) or whole brain (**F**) but increased ratio of apo-Tf increases release into the brain (**E**). Notably, the MVs contain substantially more ^55^Fe than the entirety of the brain, supporting the role of MVs in regulating iron release. n = 5 to 6 for all conditions, means of biological replicates ± SD were evaluated for statistical significance using one-way ANOVA with Tukey’s posttest for significance

The regulation of ^55^Fe-Fth1 uptake by apo- and holo-Tf infusion was examined next. In male mice, ^55^Fe-Fth1 levels in the MVs (Fig. 3a), parenchyma (Fig. 3b), and total whole brain uptake (Fig. 3c) were not significantly different in response to either holo or apo Tf infusion. Similarly, in female mice, ^55^Fe-Fth1 levels in the MVs (Fig. 3d) or whole brain (Fig. 3f) were unaltered with the respective infusions, however, parenchymal levels, as an indicator of Fe released into the brain (Fig. 3e) from the MVs, increased with infusion of apo-Tf by about 43% compared to sham but with considerable variability. Thus, the results were not statistically significant. As was the case with Tf delivered iron Fth1 delivered iron was 50% higher in the MVs than that in the parenchyma for both sexes.

### Iron uptake is strongly carrier protein- and sex-dependent

**Fig. 4:**
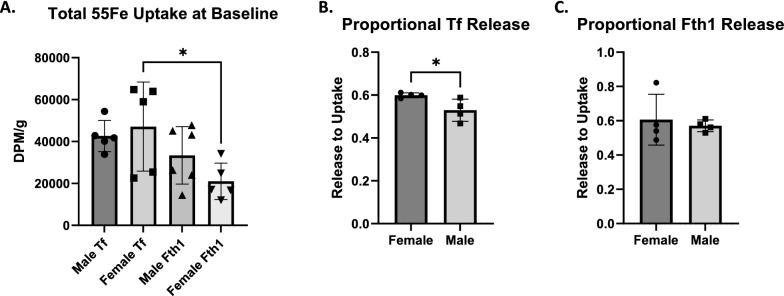
Differences in baseline iron uptake. When pooling the ^55^Fe present in both MV and brain parenchyma fractions (**A**), females take up significantly more ^55^Fe when bound to Tf than Fth1. Of note, the variability of ^55^Fe-Tf uptake and release in females is substantial. The coefficient of variability of the sham condition in females is 45.09%. The corresponding coefficient of variability of this condition in males is 17.43%. When further exploring the proportion of ^55^Fe-Tf that is released into the brain to the amount that is taken up into the MVs, females release significantly more of the iron the MVs take up compared to males (**B**). There was no difference between males and females on the proportion of ^55^Fe-Fth1 release to uptake (**C**). n = 5 to 6 for all conditions, means of biological replicates ± SD were evaluated for statistical significance using one-way ANOVA with Tukey’s posttest for significance for A. Proportions of release to uptake for each infusion condition were calculated and plotted, means ± SD were evaluated for statistical significance using unpaired t-test for significance for B and C. * p < 0.05

Next, baseline differences in total iron uptake between sexes and carrier proteins were established by pooling the ^55^Fe uptake into the MVs and brain parenchyma (Fig. 4a) from the sham control groups. Males had little difference in total brain ^55^Fe uptake whether bound to Tf or Fth1, whereas females took up 55% more iron when bound to Tf compared to Fth1 (*p < 0.05). On completing this analysis, it became apparent that there was a noticeable difference in variability of ^55^Fe-Tf total uptake between males and females. Therefore, the coefficient of variation, which is the ratio of the standard deviation to the mean, was determined for ^55^Fe-Tf uptake in sham groups in both sexes. The coefficient of variation was 17.43% in males and 45.09% in females. This level of variance in females suggested the existence of a confounding variable. The proportion of ^55^Fe released into the brain parenchyma to the ^55^Fe in the MVs was also compared between the sexes. In females the proportion of parenchymal ^55^Fe-Tf to MV ^55^Fe-Tf was significantly higher (Fig. 4b, *p < 0.05) compared to males. When bound to Fth1, the proportion of parenchymal ^55^Fe between males and females was not different (Fig. 4c).

### Reduction of circulating estrogen does not impact ^55^Fe-Tf uptake regulation

**Fig. 5:**
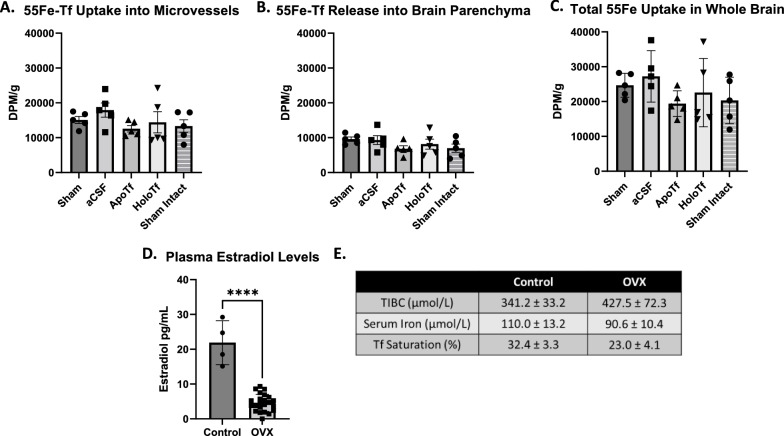
OVX on ^55^Fe-Tf brain uptake in females. Samples are reported as DPM per gram of brain tissue. After removing circulating estrogen via OVX, female mice still do not show changes in ^55^Fe-Tf uptake (**A**), release (**B**), or total uptake (**C**) across infusion conditions. Intact sham mice were included as a control. The coefficient of variability of ^55^Fe-Tf uptake in sham conditions was 16.15%. The levels of plasma estradiol levels were determined to confirm the success of the OVX in all mice used (**D**). The TIBC of plasma is higher in OVX mice while the Tf saturation percentage and serum iron are lower when compared to control (**E**). n= 5 for all conditions, means of biological replicates ± SD were evaluated for statistical significance using one-way ANOVA with Tukey’s posttest for significance for** A** and** B**. Means ± SD were evaluated for statistical significance using unpaired t-test for significance for** C** and** D**. **** p<0.0001

To determine whether the variation for the female data was related to the circulating estrogen we performed ovariectomies (OVX) on 2-month-old female mice. We hypothesized that removal of estrogen would reduce variability and result in female ^55^Fe-Tf uptake and regulatory pattern similar to the males. Two weeks after the OVX surgery, serum was isolated from the blood of the mice and confirmed their reduced estradiol levels (Fig. 5d). Three-month-old OVX mice displayed ^55^Fe-Tf uptake by MVs (Fig. 5a) and release into the parenchyma (Fig. 5b) but the patterns of uptake was unaltered by apo- or holo-Tf infusion compared to sham. Intact female mice were included to demonstrate that ^55^Fe was taken up in similar amounts in the OVX mice to the intact mice. However, the coefficient of variation of the total ^55^Fe uptake in the sham condition was 16.15% for the OVX group, which was more comparable to male variance (17.43%) than intact females (45.09%). We further analyzed the serum isolated from the mice by examining the total iron binding capacity (TIBC) and serum iron levels (Fig. 5e). TIBC was higher in OVX mice (427.5 µmol/L) compared to intact control mice (341.2 µmol/L). Serum iron was lower in OVX mice (90.6 µmol/L) compared to the control (110.0 µmol/L). Lastly, Tf saturation percentage was decreased in OVX (23.0%) compared to control (32.4%). These measures indicate the OVX produced a mild systemic iron deficiency but did not result in differences in brain iron uptake patterns or response to infusion of apo- or holo-Tf.

## Conclusions

The objective of this study was to determine the regulation of Fth1- and Tf-bound iron uptake into the brain by apo- and holo-Tf in vivo. In pursuit of this aim, we discovered significant sex differences in the regulation of iron uptake mediated by these two proteins. The results of this study have demonstrated that the ratio of apo- to holo-Tf in the CSF regulates Tf-bound brain iron uptake in males, but not in females in this model. However, there was significant variation in ^55^Fe-Tf uptake in females. To address these differences, we performed ovariectomies aimed to determine if reducing circulating estrogen would enable the regulatory response to apo- and holo-Tf infusions that were seen in males. We found that reducing peripheral estrogen did not change the lack of response of ^55^Fe-Tf uptake into MVs or release into the brain parenchyma following infusion of apo- or holo-Tf. However, the variability that had been seen in the intact females was significantly reduced to that seen in males after removal of circulating estrogen. Additionally, delivery of Fth1 bound iron was not responsive to the infusion of apo- or holo-Tf in the CSF of either males or females. A particularly notable finding in this study was that MVs contained significantly more of the injected iron regardless of the delivery protein than the brain parenchyma even though the MVs account for only 2% of the total brain cells [10]. This finding further establishes our position that the ECs serve as a reservoir for iron for subsequent regulated release into the brain. Previous studies reporting on uptake of iron or other nutrients have rarely differentiated what is in the microvasculature versus what has entered the brain parenchyma. Furthermore, our data demonstrate that acquisition of brain iron is dependent on carrier protein and sex.

Previously, we and others have postulated the concept of regulation of iron release to the brain by endothelial cells of the BBB in cell culture models [13–16, 24, 25]. For example, Simpson et al. demonstrated that CSF from iron deficient monkeys, as well as conditioned media from iron chelated astrocytes, increased iron release from bovine retinal endothelial cells [16] in a bi-chamber model of the BBB. Moreover, our group previously showed, using iPSC-derived ECs in a simulated BBB model, that exposure to apo-Tf resulted in increases in both ^59^Fe-Tf and ^59^Fe-Fth1 transport from apical to basal chambers, whereas incubation with holo-Tf decreased their transport [14]. In vitro conditions simulating brain iron deficient environments have repeatedly resulted in increased iron transport across the BBB [16, 26, 27]. However, until now, the demonstration of in vivo regulation was lacking. Our in vivo data from male mice support regulated release of iron from ECs forming the MV and suggest that the brain uses apo- and holo-Tf to relay its iron status to ECs, which in turn release more or less iron in response. An example of how this feedback can occur in situ is that, following iron uptake by neurons and astrocytes, these cells release apo-Tf into the extracellular fluid [28, 29]. Thus, areas of greater energetic activity can regionally signal for increased iron release from MVs. Our data address for the first time local regulation of brain iron uptake in response to iron utilization and help explain the findings of Beard et al. who demonstrated that brain iron uptake differs in various regions [30].

The role of Fth1 as an iron delivery protein to the brain is a relatively new concept with great implications as it binds nearly 2000 times more iron than Tf [31]. It has been reported that Fth1 can replace Tf as the iron delivery protein for oligodendrocytes [32] and ECs [14]. Fth1 is a substantial iron contributor to the brain during development, as up to postnatal day 22, mice take up significantly more Fth1 bound iron than Tf bound iron into the brain [18]. In previous in vitro studies, the iron status of Tf in the basal compartment of the BBB model impacted the amount of Fth1-bound iron that was transported across the ECs [14]. However, in this in vivo study, we did not see any significant differences in Fth1 bound iron uptake into MVs or release from the MVs into the brain parenchyma following infusion of apo- or holo-Tf. In females, the infusion of apo-Tf did result in a two-fold increase in iron release into the brain compared to sham control. Although this difference did not reach statistical significance, the Cohen’s *d* effect size between sham control and apo-Tf is 0.65, indicating a moderate effect. The absence of statistical significance was likely due to the variability in the different groups. Thus, the data suggest that Fth1 delivered iron is responsive to CSF iron deficiency in females.

Our experimental design and data interpretation is built on the premise that the infused apo- and holo-Tf exits the ventricles and exchanges with the extracellular fluid in the brain parenchyma. Intracerebroventricular (ICV) injection of iron transporter proteins has been well established by a number of foundational studies [33–36]. Moos and Morgan demonstrated that 24 h after a single ICV injection of [^125^I]Tf, up to 10% is present in the brain and 5% is still present in the CSF, while ^59^Fe was deposited past the ependyma cells near the injection site [33]. Moos further demonstrated that labeled transferrin diffused in the vicinity of the injection, reaching past the ependyma cells to neurons and glia, as well as areas along the subarachnoid space ]^34^]. Similarly, Brightman observed the rapid diffusion of ferritin after ICV injection in as little as 10 min, with further distribution into the brain tissue as time increased. More contemporary studies have reported similar ICV protein dynamics [35, 36]. Iliff et al. infused various tracer molecules into the lateral ventricle to map their distribution into the brain. Within 30 min, a 3 kDa molecule and a 2000 kDa molecule penetrated 50% and 25%, respectively, of the brain near CSF compartments [37]. Given that these models used a single injection to observe significant diffusion, our model of a steady state infusion that continuously delivers apo- or holo-Tf into the ventricles would be expected to show at least similar degrees of diffusion.

In a few experiments we conducted, infusion of aCSF alone increased iron uptake into MVs and release into the brain. Based on our calculations, the 0.25 µL/h infusion rate would have resulted in an approximately 1% dilution of total CSF and, thus, should have minimal effect on endogenous Tf levels given the complete turnover of CSF every 1.8 h in the mouse [38]. It is possible that in the less than 1 µL volume of the mouse lateral ventricle [39] this initial dilution is locally greater and may promote a regional iron uptake. Regardless, the observation that small amount of apo-Tf or aCSF increases the uptake of transferrin-bound iron to the brain MVs and subsequent release into the parenchyma underscores how exquisitely fined tuned the signaling from the brain extracellular fluid to the MVs regarding iron status is.

Significant sex differences were detected in baseline (sham control group) iron uptake between Tf and Fth1. Female mice took up significantly more iron bound to Tf than to Fth1, while there was no statistically significant difference in iron uptake by either delivery protein in males. There was an increased proportion of Tf-bound iron released into the brain in females relative to males, indicating that iron was more rapidly transported from the MVs to the parenchyma. The differences in baseline uptake would suggest differences in iron levels in the brain but studies have shown there is little to no difference in total brain iron levels between males and females [40, 41]. These studies, however, largely fail to examine the process of iron accumulation. Brain iron accumulation was addressed by Duck et al., who showed that 24 h after injecting mice with ^59^Fe-Tf, males and females had the same amount of iron uptake; however, after five days post injection, females took up significantly more ^59^Fe in to their brains than males [20]. Combined with our data presented herein, these findings indicate that females have more iron uptake over time than males. More iron accumulation by females compared to males would be consistent with increases in myelin turnover [42] and dopamine synthesis [43, 44] reported in females; both processes are dependent on iron as a co-factor [45, 46]. The constant utilization of iron for these metabolic processes likely leads to an increased requirement of iron uptake into the brain which seems to be predominantly met by regulation of Fth1. This idea is also consistent with the observation in females that Tf delivered iron in not responsive to the infusion of apo- and holo-Tf. Future studies to decipher how differences in metabolic needs impact female brain iron uptake are needed.

In conclusion, this study is the first demonstration of in vivo regulation of brain iron uptake into MVs and subsequent release into the brain parenchyma by apo- and holo-Tf. Moreover, we have identified striking sex differences in the baseline uptake and regulation of iron uptake for both Tf and Fth1. Understanding the sex differences and differences in Tf versus Fth1 delivered iron is crucial for clinical translation of these studies for the treatment of brain iron dysregulation and use for drug delivery efforts.

## Abbreviations

BBB: Blood–brain barrier
ECs: Endothelial cells
Tf: Transferrin
Fth1: Ferritin heavy chain
aCSF: Artificial cerebrospinal fluid
MVs: Microvessels
OVX: Ovariectomy
